# Epileptic focus localization using transfer learning on multi-modal EEG

**DOI:** 10.3389/fncom.2023.1294770

**Published:** 2023-11-23

**Authors:** Yong Yang, Feng Li, Jing Luo, Xiaolin Qin, Dong Huang

**Affiliations:** ^1^Chongqing Institute of Green and Intelligent Technology, Chinese Academy of Sciences, Chongqing, China; ^2^Chengdu Institute of Computer Application, Chinese Academy of Sciences, Chengdu, China; ^3^Chongqing School, University of Chinese Academy of Sciences, Chongqing, China; ^4^Department of Neurology, The First Affiliated Hospital of Chongqing Medical University, Chongqing, China

**Keywords:** epileptic focus localization, multi-modal EEG, transfer learning, adversarial training, patient-independent

## Abstract

The standard treatments for epilepsy are drug therapy and surgical resection. However, around 1/3 of patients with intractable epilepsy are drug-resistant, requiring surgical resection of the epileptic focus. To address the issue of drug-resistant epileptic focus localization, we have proposed a transfer learning method on multi-modal EEG (iEEG and sEEG). A 10-fold cross-validation approach was applied to validate the performance of the pre-trained model on the Bern-Barcelona and Bonn datasets, achieving accuracy rates of 94.50 and 97.50%, respectively. The experimental results have demonstrated that the pre-trained model outperforms the competitive state-of-the-art baselines in terms of accuracy, sensitivity, and negative predictive value. Furthermore, we fine-tuned our pre-trained model using the epilepsy dataset from Chongqing Medical University and tested it using the leave-one-out cross-validation method, obtaining an impressive average accuracy of 90.15%. This method shows significant feature differences between epileptic and non-epileptic channels. By extracting data features using neural networks, accurate classification of epileptic and non-epileptic channels can be achieved. Therefore, the superior performance of the model has demonstrated that the proposed method is highly effective for localizing epileptic focus and can aid physicians in clinical localization diagnosis.

## 1 Introduction

Epilepsy is a worldwide nervous system disease caused by sudden abnormal discharges of nerve cells in the brain. According to statistics, 70 million people worldwide suffer from epilepsy. Clinical manifestations of epileptic seizures include impaired consciousness, limb spasms, urinary incontinence, frothing, and other symptoms. Although short-term epileptic seizures have minimal impact, frequent long-term seizures severely affect patients’ physical, mental and intellectual health (Kwan and Brodie, 2000; Rakhade and Jensen, 2009; Rasheed et al., 2021).

The characteristics of EEG (electroencephalogram) data during epileptic seizure period are related to the original localization and the cause of epilepsy. Different nervous system diseases or brain conditions can cause various epileptic seizures (Babb et al., 1987; Fisher et al., 2017). In the treatment of epilepsy, around 1/3 of patients with intractable epilepsy are drug-resistant. Therefore, precise localization of the epileptic focus during presurgical assessment is necessary for the successful resection of epileptic focus.

There are four clinical methods for epileptic focus localization, including observing clinical symptoms, analyzing fMRI (functional magnetic resonance imaging) data, examining sEEG (scalp electroencephalogram) signal, and studying iEEG (intracranial electroencephalogram) signal. Each method has its advantages and limitations. Observing clinical symptoms is the most direct method but can only localize the functional brain areas. Analyzing fMRI data is expensive and has low temporal resolution. Moreover, if the seizures of epileptic patients are not caused by structural brain lesions, this method will not be able to accurately localize the epileptic focus (Morgan et al., 2004; Stufflebeam et al., 2011; Zhang et al., 2015). Examining sEEG signal is widely used in the detection and prediction of epilepsy (Zhang et al., 2021; Wan et al., 2023a; Yang et al., 2023a,b). This method is non-invasive and has high temporal resolution, but requires expert judgement with a long period of time and the judgement by different physicians may vary. Furthermore, electrodes are implanted in the appropriate target areas of the brain for iEEG signal acquisition and analysis, which is costly, complex, and carries a risk of infection, etc.

Patient-independent methods, which involve joint training with data from multiple patients, face challenges in eliminating significant differences between patients (mainly caused by multiple factors such as physical condition, pathogenesis, seizure intensity, seizure type, etc.). Moreover, the sEEG and iEEG signals are multi-modal data with significantly different characteristics. sEEG, or scalp EEG, is severely attenuated by the skull, leading to signals that are not an accurate representation of the region due to volume conduction effects. iEEG, or intracranial EEG, offers high quality signals that truly reflect the activity of the region. Combining the advantages of sEEG and iEEG data offers a promising approach for epileptic focus localization.

The main contributions of our study can be summarized as follows:

(1) In the pre-trained model, the style-feature randomization module and the domain adversarial network were introduced to enhance the generalization ability of the model, and achieving the optimal test results on the Bern-Barcelona dataset and the Bonn dataset;
(2) We have proposed a novel transfer learning method for epileptic focus localization, which can make use of the Bern-Barcelona dataset to pre-train the model. Then, we fine-tuned this pre-trained model with the epilepsy dataset from Chongqing Medical University, and conducted sufficient experiments to validate the practical applicability value of our method.

## 2 Related works

So far, a number of epileptic focus localization technologies have been developed, primarily transforming the epileptic focus localization problem into a classification task. For example, Chen et al. (2017) used discrete wavelet transform (DWT) to extract feature metrics such as Max, Min, Mean, STD, Skewness of wavelet coefficients at all levels, achieving an accuracy of 83.07% on sym6 wavelet coefficients. Daoud and Bayoumi (2020) proposed a method based on semi-supervised learning, achieving an accuracy of 93.21% on the Bern-Barcelona dataset. Zhao et al. (2020) extracted the entropy features of six frequency bands, used STFT to extract time-frequency features for EEG, and combined two features into a CNN network for feature extraction and classification, achieving an accuracy of 88.77%. Zhao et al. (2021) combined entropy, STFT, and 1D-CNN, achieving an accuracy of 93.44%. Sui et al. (2021) proposed TF-HybridNet, incorporating a 1D convolutional network and STFT for time-frequency feature extraction, achieving an accuracy of 94.3%.

In addition, the characteristics of EEG signal offer valuable information for the localization of epileptic focus. Staljanssens et al. (2017) used brain functional connectivity metrics to calculate weighted adaptive orientation transfer functions, achieving an accuracy of 88.6% on the University Hospital of Geneva epilepsy dataset. Amirsalar et al. (2019) used the Pierson correlation coefficient between signals in each lead to calculate the mean number of connections and connection strength, finally achieving a sensitivity of 80% on the Karunya University EEG dataset. Gunnarsdottir et al. (2022) proposed an algorithm to identify two groups of nodes (“sources” node and “sinks” node) in a resting-state iEEG network. They validated the SSI (source-sink index) in a retrospective analysis of 65 patients, achieving an accuracy of 79%.

The analysis shows that the existing method has the following disadvantages:

(1) The Bern-Barcelona dataset and the Bonn dataset only contain channel category information. (1) Therefore, they transformed the localization problem into a classification task, which does not achieve accurate epileptic focus localization;
(2) The Bern-Barcelona dataset contains only five patients, and the existing literature does not consider the negative impact of multi-patient differences;
(3) The dataset for epileptic focus localization is limited and the accuracy of epileptic focus localization is low.

Therefore, a method with low cost and high detection accuracy is needed to solve the above issues.

## 3 Materials and methodology

### 3.1 EEG data

In this study, we utilized three datasets, including the Bern-Barcelona dataset, the Bonn dataset and the Chongqing Medical University Epilepsy dataset. The Bern-Barcelona and the Bonn datasets were used for pre-training and model performance evaluation. The parameters of the pre-trained model were obtained by training with the Bern-Barcelona dataset, and the Chongqing Medical University epilepsy dataset was used for fine-tuning and testing.

#### 3.1.1 Bern-Barcelona dataset

Recordings from Department of Neurology, University of Bern, Switzerland were used as the first iEEG dataset in this study. To the best of our knowledge, this is the only open dataset that provides clear annotation on focal and non-focal signals during seizure-free periods (Ralph et al., 2012), including data from five patients with drug-resistant temporal lobe epilepsy and being the candidates of epilepsy surgery. The dataset contains 7,500 focal samples and 7,500 non-focal samples, each lasting 20 s with a sampling rate of 512 Hz, signals being filtered by a fourth-orders Butterworth bandpass filter with cutoff frequency at 0.5 and 150 Hz.

#### 3.1.2 Bonn dataset

The second iEEG dataset used in this study, obtained from the Epileptology Department of Bonn University (Andrzejak et al., 2001), consists of five sets of EEG recordings labeled A to E. Each set consists of data from five subjects. Set A represents healthy subjects with open eyes. Set B is recorded from healthy subjects with closed eyes. Set C is recorded from non-epileptogenic zone of the epileptic patients’ brain, while Set D is recorded from epileptogenic zone. Lastly, Set E represents epileptic patients during ictal period. Each set contains a total of 100 EEG segments. Each segment is 23.6 s long with a sampling rate of 173.61 Hz. The iEEG signals were filtered using fourth-order Butterworth bandpass filter with cutoff frequency at 0.5 and 85 Hz. In this study, we focus on set C and D as they represent the non-focal and focal iEEG signals, respectively.

#### 3.1.3 Chongqing Medical University epilepsy dataset

The third sEEG dataset used in this study was obtained from Chongqing Medical University, including data from six patients. To expand the sample size, we selected patients with multiple seizures. The dataset comprises 16 channels. Each sample is 20 s long with a sampling rate of 512 Hz, filtered by a fourth-order Butterworth bandpass filter with cutoff frequencies at 0.5 and 150 Hz. Details of the dataset are given in Table 1.

**Table 1:** The information of the CQMUE dataset.

Patient No.	Number of seizures	Focus channel
1	9	F4
2	12	F3
3	7	FP2
4	8	O1
5	6	FP1
6	13	C3

### 3.2 Methodology

Due to the large amount of data in Bern-Barcelona dataset, which contains 7,500 focal data and 7,500 non-focal data, we have proposed a transfer learning method to make full use of the large amount of data during the pre-trained model phase. In this approach, we utilize the CQMUE dataset to fine-tune and test the model. Notably, the Bern-Barcelona dataset includes data from five epilepsy patients, with significant differences (mainly due to multiple factors such as physical condition, pathogenesis, seizure intensity, and seizure type). If we train the model directly with multi-patient data, it will quickly lead to model underfitting. To address this issue, we implemented a style-feature randomization block, a multi-level temporal-spectral feature extraction network, and a domain adversarial network to enhance the generalization ability of the pre-trained model.

#### 3.2.1 Pre-trained model

**Figure 1:**
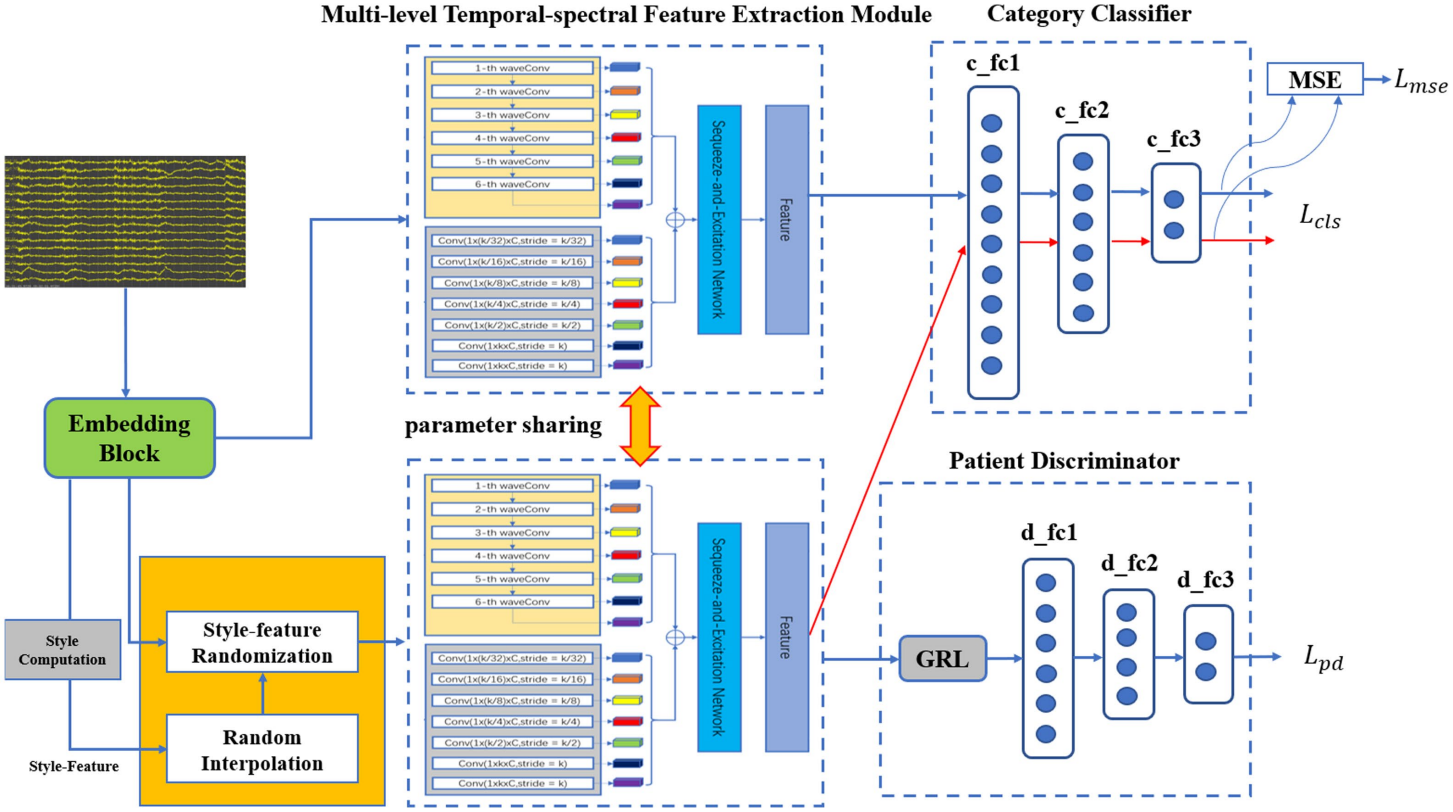
The architecture of the proposed pre-trained model.

The pre-trained model consists of an embedding block, a style-feature randomization block, a multi-level temporal-spectral feature extraction network (Hu et al., 2018; Li et al., 2020; Wan et al., 2023b), a category classifier, and a patient discriminator, as illustrated in Figure 1. The embedding block extends the data across multiple channels to enhance the discriminative properties. The style-feature randomization module disrupts data features within a training batch, enhancing the generalization ability of the model. The multi-level temporal-spectral feature extraction network utilizes temporal-spectral features to enhance feature discrimination. The category classifier completes the classification of the data. The patient discriminator employs DANN (domain-adversarial training of neural networks; Yaroslav et al., 2016) to extract the essential data features.

##### 3.2.1.1 Embedding block

Before the data is fed into the embedding block, necessary data preprocessing is required (Liu et al., 2015; Versaci et al., 2022). The embedding block, i.e., successive temporal convolution and batch normalization (BN) operations, was initially employed to derive an optimal filter band for subsequent analysis [since convolution operators are essentially equivalent to a low-pass filter (Azimi et al., 2019)]. As a result, after stacking the original data and the output embeddings with a channel-wise concatenation function, the embedding block obtained sub-band matrices that provided a subsequent network with adaptive sub-band responses and actual data. Finally, the data was fed into the multi-level temporal-spectral feature extraction module for feature extraction.

##### 3.2.1.2 Style-feature randomization

Within a training batch, the sub-band matrices are computed by the embedding block. Due to significant style-feature differences between the data of each sub-band for different patients, an enhancement of the model’s generalization ability is necessary. To achieve this, we computed $\mu_{\mathrm{nc}}\left(x\right)$ and $\delta_{nc}\left(x\right)$ across spatial dimensions independently for each sub-band (Oren et al., 2021).


(1) $$\mu_{\mathrm{nc}}\left(x\right)=\frac{1}{HW}\overset{H}{\underset{h=1}{\sum }}\overset{W}{\underset{w=1}{\sum }}x_{nchw}$$

(2) $$\delta_{nc}\left(x\right)=\sqrt{\frac{1}{HW}\overset{H}{\underset{h=1}{\sum }}\overset{W}{\underset{w=1}{\sum }}{\left(x_{nchw}-\mu_{\mathrm{nc}}\left(x\right)\right)}^{2}+\varepsilon }$$

where $X\in {\mathbb{R}}^{N\times C\times H\times W}$ , N represents the batch size, C represents the number of channels, H represents the height of the data matrix, and W represents the width of the data matrix. $x_{nchw}$ represents an element in the data matrix, $\mu_{\mathrm{nc}}\left(x\right)$ and $\delta_{nc}\left(x\right)$ represent the mean and standard deviation for each sub-band.

Then, we randomly disrupt $\mu_{\mathrm{nc}}\left(x\right)$ and $\delta_{nc}\left(x\right)$ to obtain μ(x) and σ=(x) , $x^{\prime }$ is obtained by the following equation finally.


(3) $$x^{\prime }=\frac{x-\mu \left(x\right)}{\sigma \left(x\right)}$$

where x is the sub-band matrix obtained by the embedding block.

##### 3.2.1.3 Multi-level temporal-spectral feature extraction network

To prevent deformation of the boundary data caused by zero padding in the convolution operation, the head and tail of the data are filled according to Eq. (4) (Li et al., 2020):


(4) $$x_{p}=x\left(N-\frac{R}{2}+1\right),\ldots ,x\left(N-1\right)\left|x\left(0\right),\ldots ,x\left(N-1\right)\right|x\left(0\right),\ldots ,x\left(\frac{R}{2}-2\right)$$

where | is a concatenating operator, x(i) represents the *i*-th element of input x , and R represents the parameter kernel size in the convolution operation.

To expedite calculation time, the proposed method adopted convolution operation to perform multi-level wavelet decomposition, which is defined as follows (Li et al., 2020):


(5) $$y_{A}\left(i\right)=\left(x_{p}⊗g\right)\left(i\right)=\overset{R}{\underset{r=0}{\sum }}x_{p}\left(s\times i-r\right)\times g\left(r\right)$$

(6) $$y_{D}\left(i\right)=\left(x_{p}⊗h\right)\left(i\right)=\overset{R}{\underset{r=0}{\sum }}x_{p}\left(s\times i-r\right)\times h\left(r\right)$$

where ⊗ is the convolution operation, g and h represent a pair of scaling and wavelet filter, s represents the parameter stride in the convolution operation, $y_{A}\left(i\right)$ represents the approximation (low pass) coefficients, and $y_{D}\left(i\right)$ represents the detail (high pass) coefficients.

In the multi-level temporal feature extraction module, we adopted five independent convolution, batch normalization, and empirical linear unit (ELU) operations to capture multi-level temporal feature information within different perceptual domains. The convolution kernel size is set to [S, 1], where the value of S is { k,k,k/2,k/4,k/8,k/16 }, $k=2^{6}$ , and ultimately, the temporary features ( $f_{t1}$ , $f_{t2}$ , $f_{t3}$ , $f_{t4}$ , $f_{t5}$ , $f_{t6}$ ) are derived.

In the multi-level spectral feature extraction module, we selected Daubechies order-4 (Db4) wavelet function to extract the corresponding wavelet coefficients within standard physiological sub-bands δ(0 ~ 4 Hz), θ(4 ~ 8 Hz), α(8 ~ 16 Hz), β(16 ~ 32 Hz), and γ(32 ~ 64 Hz), high-γ(65 ~ 128 Hz). Finally, the frequency features ( $f_{\delta }$ , $f_{\theta }$ , $f_{\alpha }$ , $f_{\beta }$ , $f_{\gamma }$ , $f_{high-\gamma }$ ) are derived.

To further extract discriminative feature information, the features extracted by the multi-level temporal feature extraction module and the multi-level spectral feature extraction module were combined according to the feature dimensions:


(7) $$f_{all}=\left\{\begin{array}{l} \left[f_{\delta }|f_{t1}\right],\left[f_{\theta }|f_{t2}\right],\left[f_{\alpha }|f_{t3}\right],\\ \left[f_{\beta }|f_{t4}\right],\left[f_{\gamma }|f_{t5}\right],\left[f_{high-\gamma }|f_{t6}\right] \end{array}\right\}$$

The combined features $f_{all}$ were fed into a multi-level squeeze-and-extinction network (Hu et al., 2018) to enhance discriminability of features.

##### 3.2.1.4 Category classifier

For the category classifier, the method utilized data from each channel to achieve binary classification of epileptic and non-epileptic focus channels. A 3-layer fully connected network was employed for the category classifier. We applied the CrossEntropy loss to achieve accurate classification and the MSE (Mean Squared Error) loss to minimize the output differences between source data and style-feature randomization data. The loss functions of the classification network are as follows:


(8) $$L_{cls}=\frac{1}{N}\underset{x_{i}\in D_{s}}{\sum }\left(\begin{array}{l} L\left(G_{c}\left(G_{f}\left(EB\left(x_{i}\right)\right)\right),y_{i}\right)\\ +L\left(G_{c}\left(G_{f}\left(SR\left(EB\left(x_{i}\right)\right)\right)\right),y_{i}\right) \end{array}\right)$$

(9) $$L_{mse}=\frac{1}{N}\underset{x_{i}\in D_{s}}{\sum }{\left(G_{c}\left(G_{f}\left(EB\left(x_{i}\right)\right)\right)-G_{c}\left(G_{f}\left(SR\left(EB\left(x_{i}\right)\right)\right)\right)\right)}^{2}$$

where L is the CrossEntropy loss function, EB represents the embedding block, SR represents style-feature randomization, $G_{f}$ represents the multi-level temporal-spectral feature extraction network, $G_{c}$ represents the category classifier, $y_{i}$ represents the category label, $x_{i}$ represents input samples, and $D_{s}$ represents a dataset.

##### 3.2.1.5 Patient discriminator

Since the dataset contains data from multiple patients, we have proposed a method based on the DANN (Yaroslav et al., 2016) to enhance the generalization ability of the model. Features from each patient were extracted according to the marginal distribution by the global adversarial network. The global adversarial loss function is as follows:


(10) $$L_{pd}=\frac{1}{N}\underset{x_{i}\in D_{s}}{\sum }L\left(G_{pd}\left(G_{f}\left(x_{i}\right)\right),d_{i}\right)$$

where L is the CrossEntropy loss function, $G_{f}$ represents the multi-level temporal-spectral feature extraction network, $G_{pd}$ represents the patient discriminator, $d_{i}$ represents the patient label, and $D_{s}\in D_{1}\cup D_{2}\ldots \cup D_{n}$ represents the patient sample set.

##### 3.2.1.6 Training details

We proposed an adversarial training strategy to train the loss functions jointly:


(11) $$L_{sum}=L_{cls}+\lambda_{1}\times L_{mse}-\lambda_{2}\times L_{pd}$$

Where $\lambda_{1}=0.01$ , $\lambda_{2}=0.01$ . ${\hat{\theta }}_{f},{\hat{\theta }}_{c},{\hat{\theta }}_{pd}$ are trained by a special layer called Gradient Reversal Layer (GRL). This GRL is omitted during forward propagation, and the gradient is reversed in backpropagation. Finally, we searched for the optimal parameters ${\hat{\theta }}_{f},{\hat{\theta }}_{c},{\hat{\theta }}_{pd}$ to meet the following requirements:


(12) $$\left({\hat{\theta }}_{f},{\hat{\theta }}_{c}\right)=\arg\min\underset{\theta_{f},\theta_{c}}{L_{sum}}\left(\theta_{f},\theta_{c},\theta_{pd}\right)$$

(13) $$\left({\hat{\theta }}_{pd}\right)=\arg\max\underset{\theta_{pd}}{L_{sum}}\left(\theta_{f},\theta_{c},\theta_{pd}\right)$$

where $\theta_{f}$ is the parameters of the multi-level temporal-spectral feature extraction network, $\theta_{c}$ represents the parameters of the category classifier, and $\theta_{pd}$ represents the parameters of the patient classifier.

#### 3.2.2 Model fine-tuning

The parameters of feature extraction module in the pre-trained model were frozen, and then the category classifier was fine-tuned using the CQMUE dataset. In the CQMUE dataset, only one channel is a seizure channel, causing an imbalance between the positive and negative samples. To address this issue, we have introduced the weighted CrossEntropy loss function (Rezaei-Dastjerdehei et al., 2020):


(14) $$L_{\mathrm{weight}}=-\overset{M}{\underset{c=1}{\sum }}w_{c}y_{c}\log\left(p_{c}\right)$$

where $w_{c}$ is the weight parameter for each category within the dataset, $w_{c}=\frac{N-N_{c}}{N}$ , N represents the total number of samples in the dataset, $N_{c}$ represents the number of samples for each category within the dataset. M represents the number of categories, $p_{c}$ represents the model output probability, and $y_{c}$ represents the label for each category.

**Figure 2:**
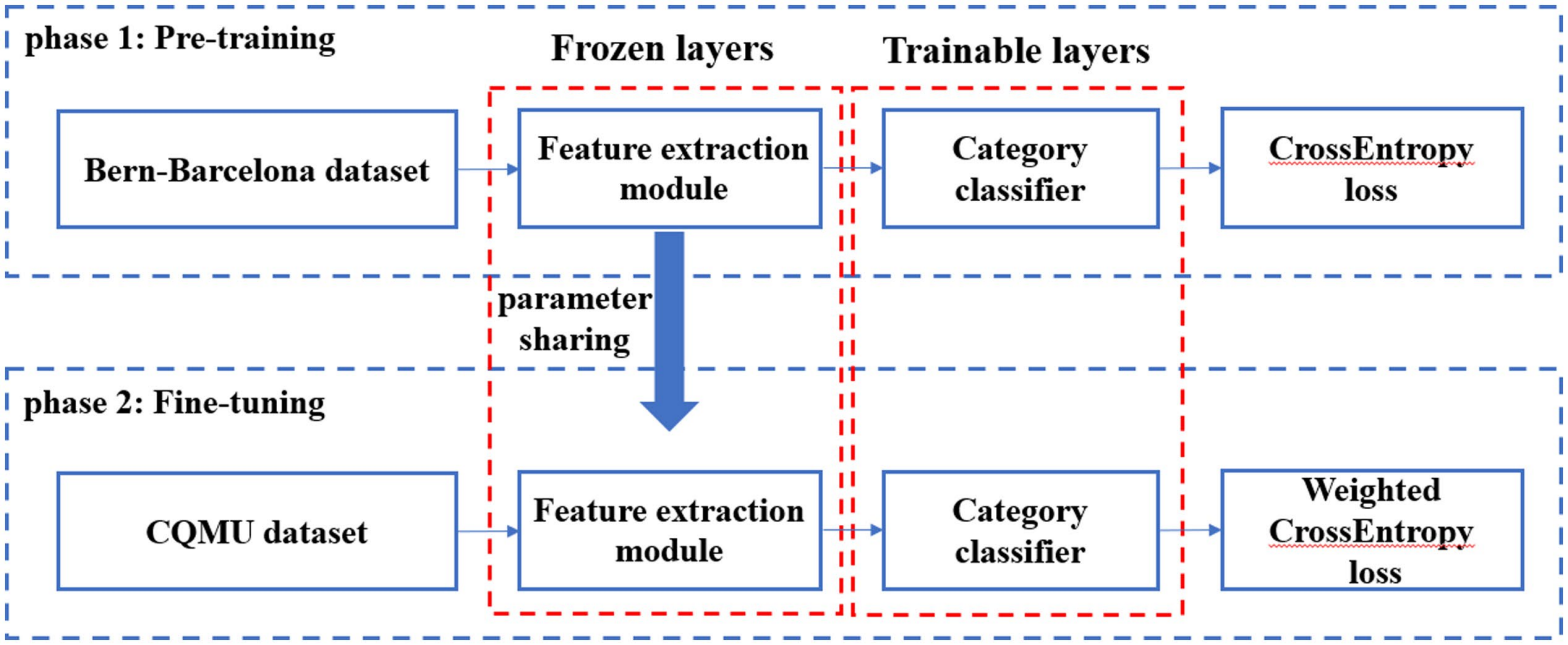
Transfer learning model.

In the fine-tuning phase, the CrossEntropy loss function must be replaced with the weighted CrossEntropy loss function. The parameters of the feature extraction module were frozen, while the parameters of the category classifier were trainable. The transfer learning model is shown in Figure 2.

#### 3.2.3 Result visualization

**Figure 3:**
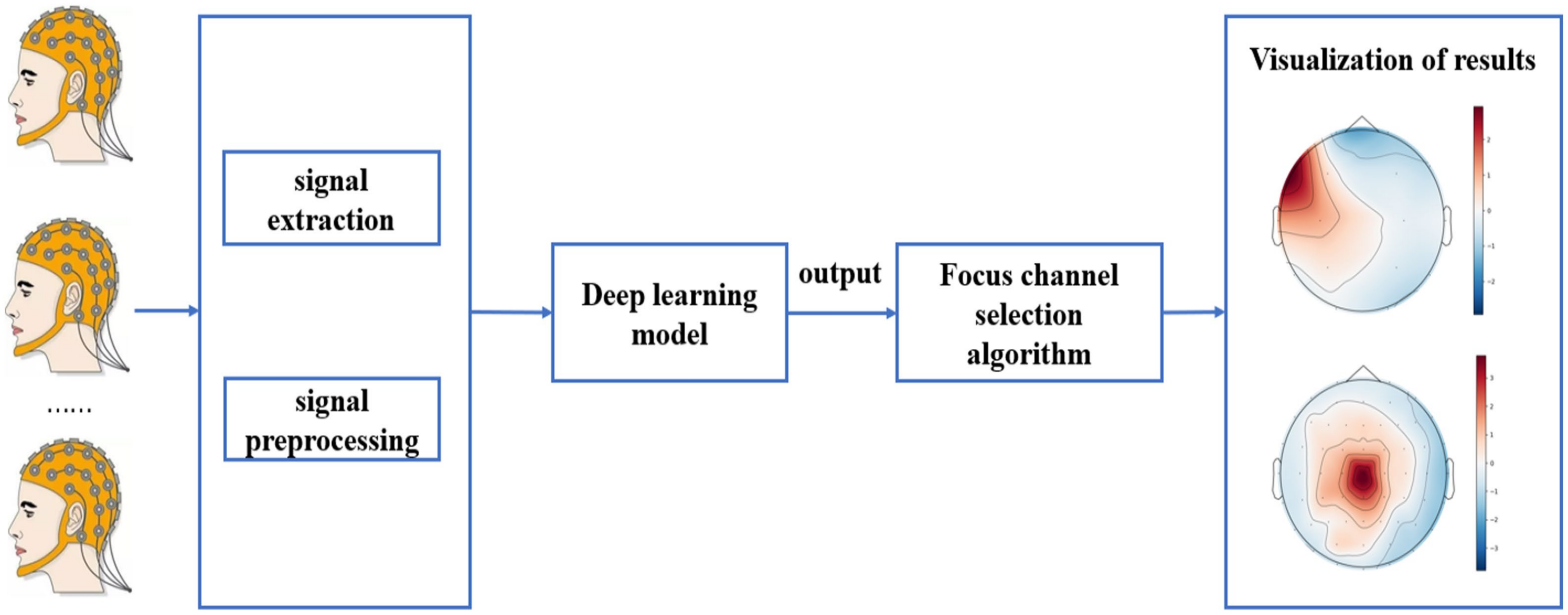
Test procedure on the CQMUE dataset.

For the test data, first the output probability of each channel was calculated, then the channel with the highest output probability was selected as the epileptic focus channel, and finally the output probability of each channel was visualized by whole brain topography. The test procedure is shown in Figure 3.

### 3.3 Evaluation

To evaluate the pre-trained models, a 10-fold cross-validation was performed on the Bern-Barcelona dataset and the Bonn dataset. All data were randomly scrambled and divided into 10 parts, one part of which was used for testing and the others for training.

Moreover, testing our model through the leave-one-out cross-validation method has validated the robustness of our approach on the CQMUE dataset, i.e., data from one person are used for testing and data from another person are used for fine-tuning the model. The training results were averaged to obtain the final test results.

#### 3.3.1 Experimental parameters

The experimental environment included Windows 10 operating system, Python 3.7.4 as the program language, and Pytorch (version 11.1) as the deep learning framework. The graphics card used was: GeForce RTX 3060.

The training epoch was set to 100 times and the batch size was set to 100. The loss function consists of the CrossEntropy loss function, the weighted CrossEntropy loss function and the MSE loss function. The model adopted the Adam optimizer, and the learning rate was set to 0.0005. All parameters were optimized using grid search.

#### 3.3.2 Evaluation metrics

The experiment employed accuracy (ACC), sensitivity (SN), specificity (SP), positive predictive value (PPV) and negative predictive value (NPV) to quantify the performance of the proposed method (Chen et al., 2017).


(15) $$ACC=\frac{TP+TN}{TP+FN+FP+FN}$$

(16) $$SN=\frac{TP}{TP+FN}$$

(17) $$SP=\frac{TN}{TN+FP}$$

(18) $$PPV=\frac{TP}{TP+FP}$$

(19) $$NPV=\frac{TN}{TN+FN}$$

where TP, TN, FP, and FN are true positive, true negative, false positive and false negative, respectively.

## 4 Experiments and discussions

### 4.1 Overall comparison

In this section, we computed a number of performance metrics such as accuracy, sensitivity, specificity, positive predictive value (PPV) and negative predictive value (NPV) on the Bern-Barcelona and Bonn datasets respectively, to evaluate our pre-trained model. The classification accuracy of the proposed method was 94.50% when applied to the Bern-Barcelona dataset, while it was 97.50% when applied to the Bonn dataset. The high accuracy was due to the use of convolutional layers, style-feature randomization, squeeze-and-extinction network, and domain adversarial. The robustness of our approach has been validated via the 10-fold cross-validation method.

For an additional evaluation of our method, we performed a comparison experiment on the same dataset. Table 2 shows the results on the Bern-Barcelona dataset, and Table 3 shows the results on the Bonn dataset.

**Table 2:** Results on the Bern-Barcelona dataset.

Ref.	Method		Data selection	ACC	SN	SP	PPV	NPV
	Feature extraction	Classification						
Lima and Coelho (2011)	DWT	SVM	10-fold CV	80.90%	80.40%	81.40%	81.20%	80.60%
Chen et al. (2017)	DWT	SVM	Leave-one-out	83.07%	83.05%	83.09%	83.09%	83.05%
Sharma et al. (2015)	EMD + Entropy	SVM	10-fold CV	87.00%	90.00%	84.00%	87.20%	90.50%
Zhao et al. (2018)	Entropy	FCNN	10-fold CV	81.50%	N/A	N/A	N/A	N/A
Zhao et al. (2018)	Entropy	CNN	10-fold CV	83.00%	N/A	N/A	N/A	N/A
Zhao et al. (2020)	STFT+ Entropy	FCNN	10-fold CV	88.77%	N/A	N/A	N/A	N/A
Zhao et al. (2021)	STFT+ Entropy+1D-CNN	FCNN	10-fold CV	93.44%	N/A	94.38%	N/A	N/A
Daoud and Bayoumi (2020)	DCAE	MLP	10-fold CV	93.21%	90.50%	95.92%	95.68%	90.99%
Sui et al. (2021)	TF-HybridNet	FCNN	10-fold CV	94.30%	94.30%	94.30%	94.30%	94.30%
Ours	Multi-level temporal-spectral feature extraction	FCNN	10-fold CV	**94.50%**	**95.00%**	**93.90%**	**94.20%**	**94.70%**

The bold text are to highlighted the results of the methodology presented in this paper.

**Table 3:** Results on the Bonn dataset.

Ref.	Method		Data selection	ACC	SN	SP	PPV	NPV
	Feature extraction	Classification						
Chen et al. (2017)	DWT	SVM	10-fold CV	88.00%	92.24%	83.76%	85.03%	91.52%
Daoud and Bayoumi (2020)	DCAE	MLP	10-fold CV	96.00%	93.00%	99.00%	98.90%	93.40%
Ours	Multi-level temporal-spectral feature extraction	FCNN	10-fold CV	**97.50%**	**98.00%**	**97.00%**	**97.00%**	**98.00%**

The bold text are to highlighted the results of the methodology presented in this paper.

**Figure 4:**
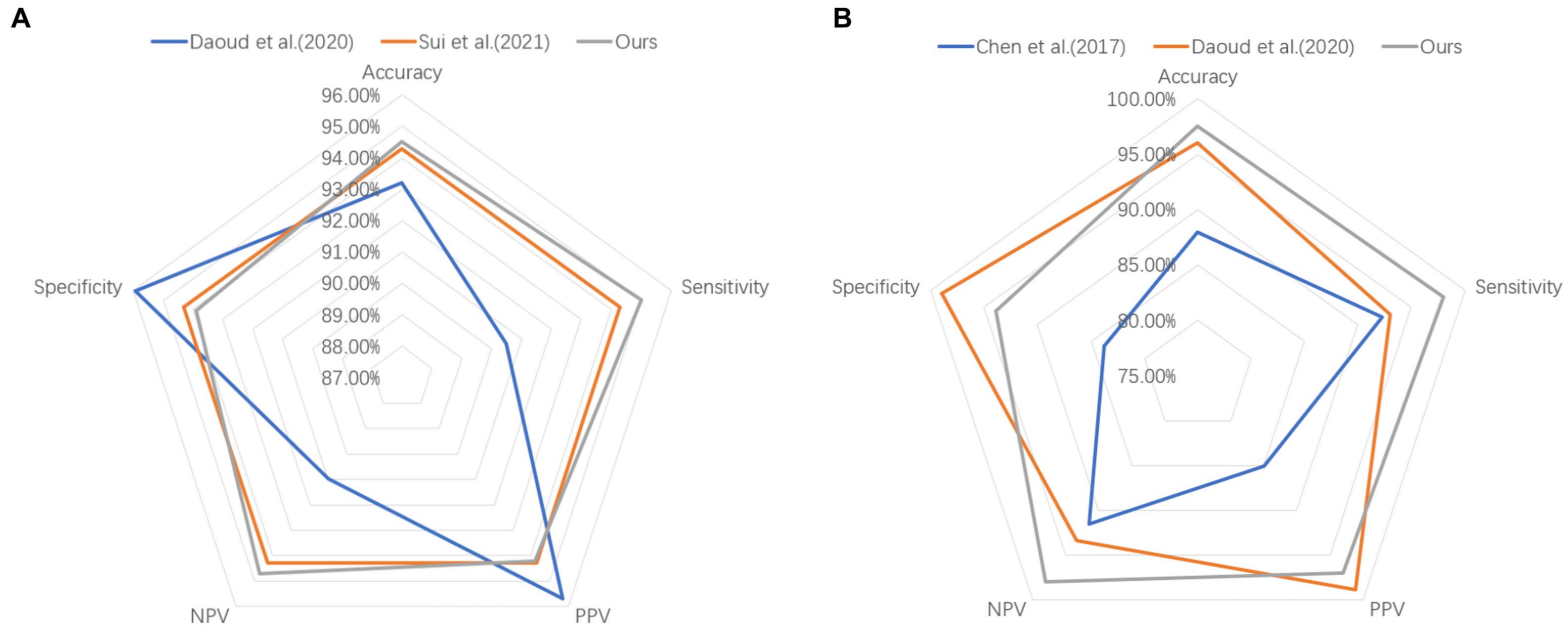
Comparison results on **(A)** Bern-Barcelona dataset and **(B)** Bonn dataset.

To better demonstrate the comparison results between our proposed method and other methods on both datasets, we adopt the radar chart, which can compare the superiority using several different indicators (accuracy, sensitivity, specificity, PPV, NPV). It is clear that our method covers a larger pentagon in both datasets, which shows that our approach outperforms previous work in localizing the epileptic focus. The radar charts are illustrated in Figure 4.

We employed the CQMUE dataset to validate the performance of the pre-trained model. The CQMUE dataset contains EEG data from only six patients, while each patient has multiple epileptic seizures. In the fine-tuning and validation experiment, we use the leave-one-out cross-validation method, i.e., data from five patients are used for model fine-tuning and data from the remaining patient are used for validation. To avoid model overfitting, we applied the method of increasing the number of fine-tuning samples, each EEG sample lasting 20 s and adjacent fragments with 90% overlap. For data from one patient used for validation, we obtained only one sample (20 s) in each epileptic seizure. Experimentally, the average accuracy of epileptic focus localization was 90.15%. The results are shown in Table 4.

**Table 4:** Test results on the CQMUE dataset.

Patient No.	ACC
1	88.90%
2	83.30%
3	85.70%
4	100.00%
5	83.00%
6	100.00%
Average	**90.15%**

The bold text are to highlighted the results of the methodology presented in this paper.

To better demonstrate the comparison between our proposed method and related literature (Amirsalar et al., 2019) on the CQMUE dataset, we can find in Table 5 that our method achieved a high accuracy of 90.15%.

**Table 5:** Performance comparison between proposed method and related literature.

Ref.	Method	ACC
Amirsalar et al. (2019)	Pierson correlation matrix	75.50%
Ours	Multi-level temporal-spectral feature extraction	**90.15%**

The bold text are to highlighted the results of the methodology presented in this paper.

### 4.2 Ablation experiments with pre-trained models

To validate the performance of the style-feature randomization module and the DANN in the pre-trained model, we performed ablation experiments on the Bern-Barcelona and Bonn datasets. We tested the performance of removing the style-feature randomization module, removing the DANN, and removing both the style-feature randomization and DANN modules, and compared them with the proposed method. Tables 6, 7 show that the performance of the model decreased after removing the style-feature randomization and DANN modules on the Bern-Barcelona and Bonn datasets, respectively.

**Table 6:** Ablation experiments on the Bern-Barcelona dataset.

No.	Method	ACC	SN	SP	PPV	NPV
1	Remove style-feature randomization	92.60%	91.90%	93.30%	93.20%	92.00%
2	Remove DANN	92.40%	92.30%	92.40%	92.40%	92.30%
3	Remove style feature randomization and DANN simultaneously	91.80%	91.10%	92.60%	92.50%	91.20%
4	Ours	**94.50%**	**95.00%**	**93.90%**	**94.20%**	**94.70%**

The bold text are to highlighted the results of the methodology presented in this paper.

**Table 7:** Ablation experiments on the Bonn dataset.

No.	Method	ACC	SN	SP	PPV	NPV
1	Remove style-feature randomization	92.50%	92.00%	93.00%	92.90%	92.10%
2	Remove DANN	92.00%	94.00%	90.00%	90.40%	93.80%
3	Remove style feature randomization and DANN simultaneously	90.00%	89.00%	91.00%	90.80%	89.20%
4	Ours	**97.50%**	**98.00%**	**97.00%**	**97.00%**	**98.00%**

The bold text are to highlighted the results of the methodology presented in this paper.

## 5 Conclusion

In this paper, we have proposed a deep learning model for the localization of epileptic focus. This method includes a pre-training phase and a fine-tuning phase. In the pre-training phase, the model adopted a multi-level temporal-spectral feature extraction model and an attention mechanism to enhance the feature extraction ability, achieving an average focus localization accuracy of 94.5% on the Bern-Barcelona dataset and 97.5% on the Bonn dataset, respectively. When compared with related methods, the experimental results have demonstrated that the pre-trained model outperforms competitive state-of-the-art baselines in accuracy, sensitivity, and negative predictive value. To validate the model’s actual performance, we fine-tuned our pre-trained model using the epilepsy dataset from Chongqing Medical University and conducted tests, obtaining an impressive average accuracy of 90.15%. Therefore, the superior performance of the model has demonstrated that the proposed method is highly effective for localizing epileptic focus. Next, we will develop a medical device that incorporates the proposed method to assist physicians’ clinical localization diagnosis of epileptic focus.

## Data availability statement

The datasets presented in this study can be found in online repositories. The names of the repository/repositories and accession number(s) can be found at: https://www.upf.edu/web/ntsa/downloads/-/asset_publisher/xvT6E4pczrBw/content/2012-nonrandomness-nonlinear-dependence-and-nonstationarity-of-electroencephalographic-recordings-from-epilepsy-patients.

## Ethics statement

The studies involving humans were approved by the First Affiliated Hospital of Chongqing Medical University. The studies were conducted in accordance with the local legislation and institutional requirements. Written informed consent for participation was not required from the participants or the participants’ legal guardians/next of kin in accordance with the national legislation and institutional requirements.

## Author contributions

YY: Methodology, Software, Writing – original draft. FL: Data curation, Resources, Writing – original draft. JL: Data curation, Resources, Writing – original draft. XQ: Supervision, Writing – review & editing. DH: Supervision, Validation, Writing – review & editing.
